# Dirty Thermometers

**Published:** 1898-02

**Authors:** 


### Dirty Thermometers.

Did you ever think how it would repulse you to have a thermometer which had been removed from another patient's mouth and not cleansed, thrust into your mouth ? Then always call for a glass of water and cleanse your thermometer *in the presence* of your patient, that he may know you have not been neglectful. Most patients are too polite to say anything, but they do a great deal of thinking, which may not result to your advantage.—*Peoria Medical Journal.*

It is to be hoped that dentists who read this will make a note of it.—Ed. Reg.

